# The One Health teaching clinic as an interprofessional education learning environment: an Austrian community case study of veterinary PhD students' competency development through co-design with pre-service science teachers

**DOI:** 10.3389/fvets.2026.1781542

**Published:** 2026-08-28

**Authors:** Ulrich Hobusch, Sascha Krebser, Franziska Messenböck, Sebastian Linnartz, Veronika Fraisl, Eva-Maria Bartl, Damien Ernst, Pia Maria Saria, Kristina Schmidhofer, Simona Winkler, Christine Aurich, Martin Scheuch

**Affiliations:** 1 Centre for Biology Teacher Education, Karl-Franzens-University, Graz, Austria; 2 University College of Agricultural and Environmental Education, Vienna, Austria; 3 Institute for Biology Education, Justus Liebig University, Giessen, Germany; 4 Biology Education, Ruhr University Bochum, Bochum, Germany; 5 Unit for Ruminant and Camelid Medicine, Clinical Centre for Ruminants, Camelids and Herd Health Management, Clinical Department for Farm Animals and Food System Transformation, University of Veterinary Medicine Vienna, Vienna, Austria; 6 Unit for Microbiology, Center for Pathobiology, Department for Biological Sciences and Pathobiology, University of Veterinary Medicine Vienna, Vienna, Austria; 7 Centre for Food Science, Clinical Department for Farm Animals and Food System Science, University of Veterinary Medicine Vienna, Vienna, Austria; 8 Clinical Centre for Population Medicine in Fish, Pig and Poultry, Clinical Department for Farm Animals and Food System Science, University of Veterinary Medicine Vienna, Vienna, Austria; 9 Centre for Food Science, Food Hygiene and Technology, Clinical Department for Farm Animals and Food Systems Transformation, University of Veterinary Medicine Vienna, Vienna, Austria; 10 Centre for Veterinary Public Health & One Health, Clinical Department for Farm Animals and Food Systems Transformation, University of Veterinary Medicine Vienna, Vienna, Austria; 11 Small Animal Internal Medicine, Department for Small Animals and Horses, University of Veterinary Medicine Vienna, Vienna, Austria; 12 Centre for Animal Nutrition and Welfare, Clinical Department for Farm Animals and Food System Science, University of Veterinary Medicine Vienna, Vienna, Austria; 13 Messerli Research Institute, Department of Interdisciplinary Life Sciences, University of Veterinary Medicine Vienna, Vienna, Austria; 14 Unit of Pharmacology and Toxicology, Center of Biological Sciences, Department of Biological Sciences and Pathobiology, University of Veterinary Medicine Vienna, Vienna, Austria; 15 Clinical Center for Reproduction, Department for Small Animal and Horses, University of Veterinary Medicine Vienna, Austria; 16 Austrian Educational Competence Centre for Biology, University of Vienna, Vienna, Austria

**Keywords:** community case study, interprofessional education, One Health education, One Health teaching clinic, science teacher education, Synoptic Transfer Framework, veterinary doctoral training

## Abstract

One Health education increasingly requires competencies that conventional veterinary doctoral training does not systematically cultivate, including science communication with non-scientific audiences, transdisciplinary collaboration, and the ability to translate specialized research into societal and educational contexts. This community case study examines how five veterinary PhD students at the University of Veterinary Medicine Vienna experienced the One Health Teaching Clinic (OHTC), a 13-week interprofessional interprofessional teacher education course in which they collaborated with pre-service science teachers to develop classroom materials based on their respective One Health doctoral projects. The OHTC is structured through the Synoptic Transfer Framework, a Two-Eyed Seeing-informed pedagogical framework that organizes changing perspectives between scientific knowledge and lifeworld understanding. Qualitative content analysis of 114 segments from learning journals, a focus group, and videographic recordings used the NEOH One Health competency framework deductively, while segments beyond the framework were coded inductively. Three patterns were central. First, the OHTC surfaced an unexpected language adaptation challenge: veterinary doctoral students whose research communication was strongly shaped by English-language scientific discourse struggled to translate specialized knowledge into accessible German explanations for non-specialist educational audiences. Second, seven situated communication practices emerged across all five cases, including content adaptation for specific audiences, the use of analogies and visuals, adjustment of language register between scientific and everyday discourse, balancing precision and accessibility, concrete exemplification, content prioritization, and risk communication that conveyed zoonotic risk without inducing unnecessary alarm. Third, participants began to recognize educational research as a legitimate knowledge system, describing an epistemological shift from a worldview confined to “*p*-values and graphs.” Notably, 32.5% of coded material fell outside the NEOH framework. Within this beyond-NEOH material, 20 unprompted implementation proposals indicated that the co-design format activated strategic action competence not fully captured by current One Health competency models. Although the findings are exploratory and limited to a single cohort at two Austrian institutions, the study suggests that structured interprofessional co-design between veterinary researchers and education professionals can create meaningful opportunities for developing the societal engagement capacities that One Health demands.

## 1 Introduction

The One Health approach recognizes the interdependence of human, animal, and environmental health and seeks to optimize health for all sections. Therefore, the approach calls for professionals who can think, communicate, and act across disciplinary and societal boundaries (1). Yet educational demand remains only partially realized. Although One Health has become an influential framework in health and life science research, social science fields, including education, remain weakly connected to the dominant biomedical and ecological clusters of One Health scholarship [(2), Figure 4]. This disconnect is particularly striking because One Health is not only a research agenda, but also an educational and societal endeavor. Principle 10 of the Berlin Principles explicitly calls for investment in

“*educating and raising awareness for global citizenship and holistic planetary health approaches among children and adults in schools, communities, and universities*.” ((3), p. 3)

Recent contributions in *The Lancet Planetary Health* similarly emphasize that One Health education should begin early and be inclusive and holistic in scope (4). Competency frameworks for One Health professionals have been developed (5, 6), but their translation into concrete educational practice remains uneven (7).

This gap is particularly visible in doctoral training. One Health doctoral programs are well positioned to train researchers in biomedical, veterinary, environmental, and life science expertise. However, they do not systematically cultivate the societal, communicative, and collaborative capacities that One Health itself requires. Existing studies point to recurring deficiencies: limited scaffolding for cross-disciplinary teamwork (8), insufficient attention to social-ecological systems thinking (9), underdeveloped communication training for non-scientific audiences despite employer demand (10), and limited engagement with social, cultural, and environmental determinants of health (11). Other work highlights the marginal role of Indigenous knowledge systems and diverse worldviews beyond mainstream scientific perspectives in One Health training (12), the lack of a shared mental model for collaborative practice (13), the continued absence of One Health content from substantial parts of medical curricula (14), and persistent gaps in adaptive co-creation processes as well as implementation and evaluation methodologies for One Health initiatives (15, 16). Overall, these findings reveal a central educational paradox: doctoral students are trained to produce specialized One Health knowledge but receive limited structured support in making that knowledge usable across disciplinary, educational, and public contexts.

Addressing this paradox requires educational formats that move beyond disciplinary coursework and generic communication training. Interprofessional education offers one promising route because it places learners from different professional backgrounds into structured collaboration around shared problems. Such formats include problem-based learning, team-based learning, simulation-based education, case-based learning, interdisciplinary workshops, and service-learning, all of which can promote critical thinking, collaboration, and practical application (17). For One Health education, however, the challenge is not only to bring disciplines together. It is to create learning situations in which scientific expertise must be translated, negotiated, and made meaningful for audiences who work with different knowledge systems, professional responsibilities, and practical constraints.

The One Health Teaching Clinic (OHTC) was developed to address this challenge. In this 13-week interprofessional teacher education course, subject matter experts (SMEs) in human medicine, veterinary medicine, or environmental sciences collaborate with pre-service science teachers (PSSTs) to develop classroom interventions on One Health topics ranging from antimicrobial resistance to zoonotic diseases (18). The course has been running as a master-level course in pre-service science teacher education, and earlier iterations have documented the teacher education side of the OHTC (7). The present community case study shifts attention to the SME experience, focusing specifically on veterinary PhD students (VPhDs) functioning as SMEs. This focus is important because these doctoral students occupy a double position: they are advanced scientific trainees, but they are also learners when asked to communicate their research beyond their disciplinary environment within educational and public contexts.

This study therefore examines how veterinary PhD students experienced participation in the OHTC as an interprofessional learning format. More specifically, it asks how the co-design process with pre-service science teachers supported the development of One Health-related competencies, particularly in relation to communication, transdisciplinary collaboration, and societal engagement. By analyzing learning journals, a focus group, and videographic observations, the study contributes empirical insight into how doctoral One Health expertise changes when it is required to travel beyond the research setting and become educationally usable.

## 2 Context

The OHTC examined in this study took place in Vienna, Austria, and connected two institutions that usually operate in separate professional and educational domains. The University of Veterinary Medicine Vienna operates an interdisciplinary One Health doctoral school that enrolls 15 PhD students across veterinary, biomedical, and environmental research areas. The University College for Agricultural and Environmental Education (Hochschule für Agrar- und Umweltpädagogik; HAUP) prepares vocational environmental and agricultural education teachers for upper secondary schools. The OHTC brought these two settings together through a structured co-design process in which veterinary doctoral research became the starting point for classroom materials developed by pre-service science teachers.

Five VPhDs from the VetMedUni Vienna doctoral school participated in the OHTC in winter term 2024/2025. They constituted the complete OHTC cohort and one third of the doctoral school's total enrollment. Their research topics reflected the breadth of the doctoral school's One Health orientation: antimicrobial resistance in *Listeria monocytogenes* along the food chain (SME-1), the role of bovine beta-lactoglobulin in the allergy-protective farm effect (SME-2), zoonotic dermatophytosis in New World camelids and its implications for animal and human health (SME-3), *Bacillus cereus* toxin mechanisms investigated using intestinal organoids (SME-4), and the role of pigs in *Listeria monocytogenes* epidemiology (SME-5). All participants had completed at least 1 year of doctoral training. None had prior experience in formal teaching, science communication, or collaboration with education professionals.

This setting offered a particularly revealing case because it did not position the VPhDs as lecturers delivering simplified expert knowledge to passive recipients. Instead, it placed them in a reciprocal relationship with PSSTs. Two PSST groups at HAUP were assigned to each VPhD topic, meaning that each doctoral research project was translated into classroom materials twice by different teacher teams. The VPhDs therefore had to explain their research clearly enough for future teachers to understand, reconstruct, teach, and evaluate it in school contexts. At the same time, the PSSTs depended on the VPhDs to safeguard scientific accuracy and relevance. This mutual dependency made the collaboration more than an outreach exercise: it became a setting in which disciplinary expertise had to be negotiated and transformed.

A contextual feature that became unexpectedly consequential was language. In Austrian veterinary doctoral training, scientific communication takes place predominantly in English. Doctoral students read, write, present, and discuss their research mainly through English-language scientific terminology. The OHTC disrupted this routine by requiring the VPhDs to communicate complex research topics in German with non-specialist educational audiences. This shift was not a minor translation issue. It exposed a deeper challenge: participants had to move from an English-dominated academic register into accessible German explanations that could support classroom teaching. As the outcomes show, this linguistic transfer became one of the central learning experiences of the OHTC.

This Austrian interinstitutional setting therefore provided a productive site for examining One Health competency development under authentic conditions. The participating VPhDs were not simply asked to reflect on communication, collaboration, or societal relevance in abstract terms. They had to enact these competencies in a concrete co-design process with partners whose expertise, institutional location, and practical responsibilities differed substantially from their own.

## 3 Key programmatic elements

The OHTC was designed as a structured interprofessional teacher education course in which veterinary doctoral research became the starting point for educational transformation. Its central pedagogical feature was not the one-way transfer of expert knowledge from VPhDs to PSSTs, but the iterative negotiation of scientific accuracy, classroom applicability, and societal relevance. The course therefore created a setting in which One Health expertise had to be made understandable, teachable, and meaningful beyond the doctoral research environment.

### 3.1 Course design

The OHTC follows a 13-week workflow structured around asynchronous learning phases and three synchronous online meetings between VPhDs and PSSTs. Table 1 provides the full overview of the weekly sequence, including VPhD and PSST activities, pedagogical focus, and mapping to the Synoptic Transfer Framework hotspots. The course design draws on the Synoptic Transfer Framework (STF), a Western educational operationalization of Two-Eyed Seeing that organizes the transfer between scientific knowledge and lifeworld understanding through four educational hotspots: Engagement Contexts (ENG), Scientific Integrity (ISC), Scientific Application (APL), and Narrative Shaping (SNR) (7, 19). These hotspots did not function as a rigid linear sequence. Instead, they structured recurring movements between scientific expertise, educational interpretation, classroom application, and societal reflection.

**Table 1:** Structure of the One Health teaching clinic and mapping to the Synoptic Transfer Framework.

Week	Phase	VPhD activities	PSST activities	Focus	STF hotspot
0	Preparation	Prepare structured abstract foregrounding societal implications of their One Health PhD topic; select three core publications	—	Content preparation	ENG
1–2	Orientation and engagement	—	Receive One Health introduction and structured workbook; engage with assigned VPhD abstract and core publications; develop scientific concept maps	Foundation and scientific engagement	ENG
3	Meeting 1 (synchronous)	Present PhD research; support concept map iteration; verify scientific accuracy	Present and iterate concept maps based on VPhD feedback; ask clarifying questions	Shared understanding	ISC
	Post-meeting 1 reflection	Complete learning journal (timepoint 1; prompts in Supplementary material S9)	—	Individual reflection	
4–6	Didactic translation	—	Develop lesson plans and teaching materials using structured workbook and MOOCs; design small-scale evaluation study	Didactic reconstruction and elementarisation	APL
7	Meeting 2 (synchronous)	Verify scientific accuracy of lesson plans; provide feedback on evaluation designs	Present draft lesson plans, teaching materials, and evaluation study designs	Quality assurance and co-design	ISC/APL
	Post-meeting 2 reflection	Complete learning journal (timepoint 2; prompts in Supplementary material S9)	—	Individual reflection	
8–10	Classroom implementation	—	Teach co-designed lessons in schools; conduct evaluation studies; analyse data	Classroom application and practitioner research	APL
12	Meeting 3 (synchronous)	Hear classroom results; jointly reflect on what worked and what did not; discuss societal implications	Present classroom intervention results; jointly reflect on teaching experiences and societal implications	Collective reflection and narrative shaping	SNR
	Post-Meeting 3 reflection	Complete learning journal (timepoint 3; prompts in Supplementary material S9)	—	Individual reflection	
13	Mini-conference	Take notes for thematic analysis	Present evaluation findings and classroom experiences to VPhDs in a mini-conference format	Group-level reflection	SNR/ENG
13+	Joint conference presentation	Joint workshop and oral presentation at CARN DACH 2025 (see note)	Joint workshop and oral presentation at CARN DACH 2025 (see note)	Dissemination	SNR/ENG
~30	Late post-evaluation	Semi-structured focus group interview; reflect on lasting effects along OH core competencies (6)	—	Final evaluation	

The table summarizes the 13-week OHTC workflow, including VPhD and PSST activities, the pedagogical focus of each phase, and the corresponding Synoptic Transfer Framework hotspots.

VPhD, veterinary PhD student functioning as subject matter expert; PSST, pre-service science teacher. STF, Synoptic Transfer Framework (19), operationalized for One Health education in Hobusch et al. (7). Hotspots: ENG, Engagement Contexts; ISC, Scientific Integrity; APL, Scientific Application; SNR, Narrative Shaping. CARN DACH = Collaborative Action Research Network, German-speaking countries (https://carndach25.univie.ac.at/). Conference formats: (1) Workshop: “Education for Sustainable Development in the Context of One Health”; (2) Oral presentation: “Fostering Knowledge Exchange Through Collaborative Action Research in One Health-Relevant Topics.” “—”, no scheduled activity.

The course began with VPhD preparation in Week 0. Each VPhD prepared a structured abstract foregrounding the societal implications of their One Health PhD topic and selected three core publications. This phase was mapped to Engagement Contexts (ENG), because the VPhDs were asked to frame their research not only as specialized doctoral work, but also as content with relevance beyond the research setting.

Weeks 1–2 focused on orientation and engagement on the PSST side. The PSSTs received an introduction to One Health and a structured workbook, engaged with the assigned VPhD abstract and core publications, and developed scientific concept maps. This phase created the foundation for the first synchronous encounter by allowing the PSSTs to approach the doctoral research through their own emerging professional perspective as future teachers.

**Figure 1:**
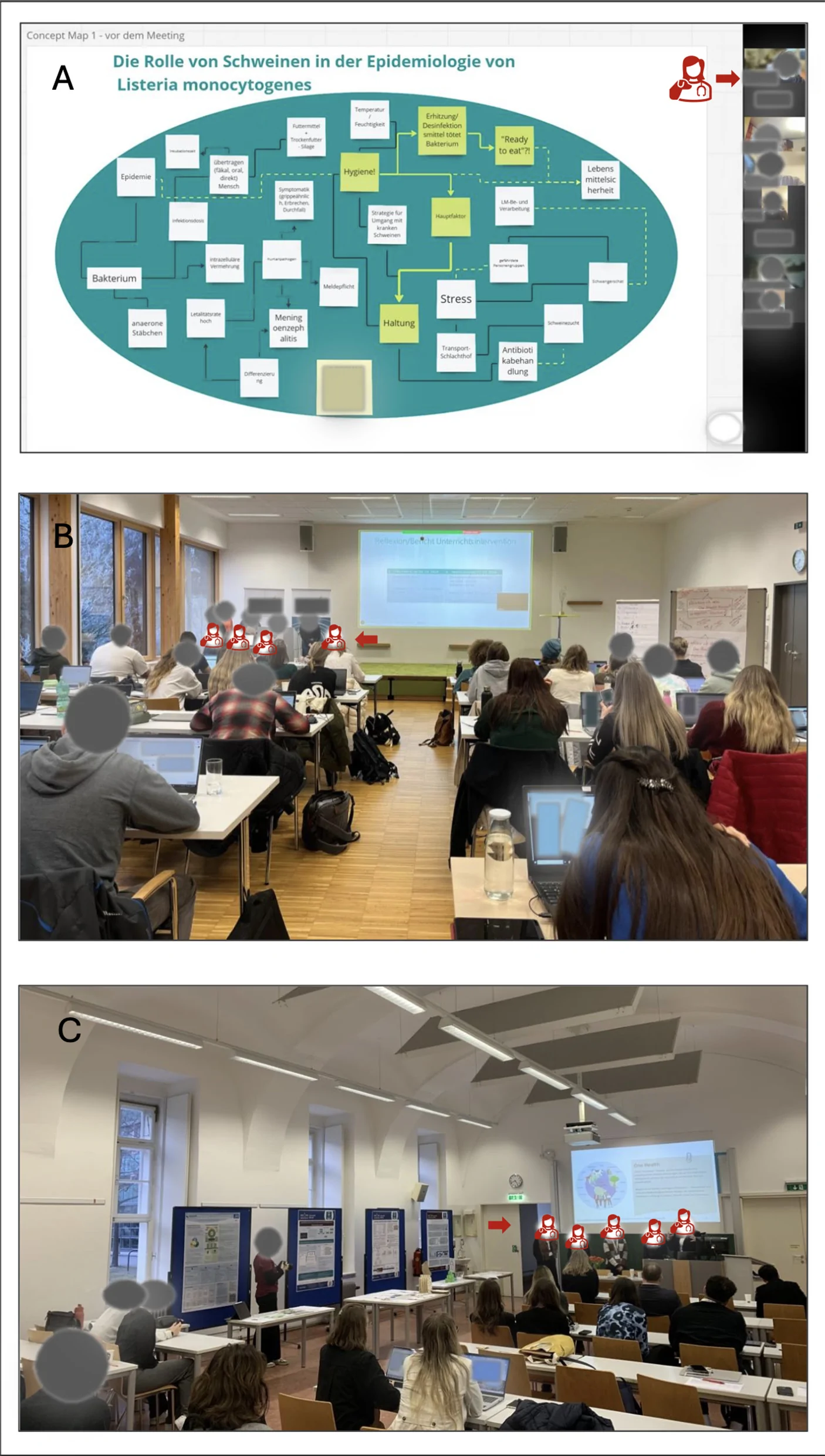
The One Health teaching clinic in practice. **(A)** Pre-service science teacher concept map on the role of swine in Listeria monocytogenes epidemiology, developed before Meeting 1; within the meeting, VPhDs help iterate the concept map to sustain scientific integrity. **(B)** Mini conference at HAUP: PSSTs present classroom intervention results to VPhDs, marked with red icons, followed by joint reflection on teaching experiences. **(C)** Joint poster presentation, oral presentation, and workshop by VPhDs and PSSTs, marked with red icons, at the CARN DACH 2025 conference, University of Vienna. All images reproduced with participant consent; faces blurred where consent for publication was not obtained.

Meeting 1 in Week 3 centered on Scientific Integrity (ISC). The VPhDs presented their PhD research, guided the revision of PSST concept maps, and verified scientific accuracy. The PSSTs presented and revised their concept maps based on VPhD feedback and asked clarifying questions. Figure 1A illustrates this phase through a PSST concept map on the role of swine in Listeria monocytogenes epidemiology, developed before Meeting 1 and iterated during the meeting with VPhD input. This meeting was the first major interdisciplinary boundary-crossing moment in the OHTC: the scientific content had to remain accurate but also become understandable enough to serve as the basis for didactic reconstruction. After Meeting 1, the VPhDs completed their first structured learning journal entry, using the prompts provided in Supplementary material S9.

Weeks 4–6 were dedicated to didactic translation. During this phase, the PSSTs developed lesson plans and teaching materials using the structured workbook and accompanying MOOCs. They also designed small-scale evaluation studies. This phase was mapped to Scientific Application (APL), because the doctoral research was reconstructed for classroom use. The focus was didactic reconstruction and elementarisation: PSSTs had to decide how complex One Health research could be transformed into teachable materials for 14–18-year-old students.

Meeting 2 in Week 7 combined Scientific Integrity and Scientific Application (ISC/APL). The PSSTs presented draft lesson plans, teaching materials, and evaluation study designs. The VPhDs verified the scientific accuracy of the lesson plans and provided feedback on the evaluation designs. This meeting functioned as a quality assurance and co-design moment: the materials were reviewed both for factual correctness and for their educational use. After Meeting 2, the VPhDs completed their second learning journal entry.

Weeks 8–10 shifted the process into classroom implementation. The PSSTs taught the co-designed lessons in schools, conducted their evaluation studies, and analyzed the resulting data. This phase was mapped to Scientific Application (APL), with a focus on classroom application and practitioner research. At this point, the doctoral research had moved beyond the university setting and had become part of concrete school-based One Health teaching.

Meeting 3 in Week 12 marked the transition to Narrative Shaping (SNR). The PSSTs presented the results of their classroom interventions, and VPhDs and PSSTs jointly reflected on what worked, what did not work, and what societal implications emerged from the teaching experiences. The focus of this meeting was collective reflection and narrative shaping. It allowed participants to consider how One Health research can be communicated, interpreted, and made meaningful in educational and societal contexts. After Meeting 3, the VPhDs completed their third learning journal entry.

In Week 13, the PSSTs presented their evaluation findings and classroom experiences to the VPhDs in a mini-conference format at HAUP. The VPhDs took notes for thematic analysis. Figure 1B shows this mini-conference setting, in which PSSTs presented their classroom intervention results to the VPhDs and engaged in group-level reflection on the teaching experiences. This phase was mapped to SNR/ENG because it combined narrative shaping with renewed engagement around how the classroom experiences could inform further work.

The collaborative process continued beyond the formal 13-week course through a joint workshop and oral presentation at the CARN DACH 2025 conference at the University of Vienna. Figure 1C documents this dissemination phase, where VPhDs and PSSTs presented the OHTC work jointly to a broader academic and professional audience. This final phase was also mapped to SNR/ENG, as it extended the OHTC from course-based co-design into broader professional and academic exchange.

The co-designed teaching materials occupied a dual role in this process. For PSSTs, they were lesson plans to be taught in schools. For VPhDs, they were simplified representations of doctoral research that had to remain scientifically accurate while becoming accessible to 14–18-year-old students. This dual function made the materials a site of productive tension. Decisions about what to include, how to handle scientific complexity and uncertainty (20, 21), and how to communicate risk responsibly (22) required repeated movement between the scientific image and the lifeworld image in the sense of the STF. The pedagogical significance of the OHTC therefore lay not only in the final classroom materials, but also in the structured negotiation through which scientific content became educationally usable.

### 3.2 Data sources and analysis

The evaluation drew on three complementary data sources: learning journals, a semi-structured focus group, and videographic recordings. Together, these sources captured individual reflection during the course, collective sense-making after course completion, and observable interaction during selected VPhD–PSST meetings.

First, all five VPhDs completed structured learning journals after the three synchronous meetings (see S9. Learning Journal Prompts). These journals produced 15 entries of varying depth. SME-1 produced the most extensive journal material, contributing 40 of the 84 journal-derived segments, while SME-4 completed two of the three planned entries. The journals captured the VPhDs' reflective experience at three points in the co-design process: after the initial negotiation of scientific understanding, after the review of teaching materials and evaluation designs, and after the presentation of classroom intervention results.

Second, a semi-structured focus group interview was conducted approximately one semester after course completion, around Week 30. In this interview, teacher education researchers from HAUP asked the VPhDs to reflect on lasting effects of the OHTC along the NEOH One Health core competency framework (6). The focus group contributed 30 coded segments. Because it took place after a temporal distance, it provided insight into what participants retrospectively identified as meaningful beyond the immediate course experience.

Third, videographic recordings of VPhD–PSST meetings were available for two of the five cases: SME-3, whose topic focused on *camelid dermatophytosis*, and SME-2, whose topic focused on *bovine beta-lactoglobulin*. These recordings yielded directly observed interaction sequences analyzed using grounded theory methodology with a separate coding system (23). The videographic data made it possible to compare participants' self-reported reflections with observable communication practices, including terminology negotiation, reformulation, and explanation strategies during the meetings.

These three data sources enabled triangulation. The learning journals captured individual reflection in real time across the semester. The focus group captured collective sense-making and lasting effects after a temporal distance. The videographies captured observable behavior during the actual meetings. This combination strengthened the analysis because the study did not rely exclusively on retrospective self-report but also included reflective and interactional data from different moments in the OHTC process.

Qualitative content analysis was conducted in accordance with Kuckartz and Rädiker (24). One science education PhD researcher and two student assistants (SK, SL, and FM) independently coded 114 identified text segments. The nine NEOH One Health competencies (6) were used as deductive categories. Segments that did not fit the framework were coded inductively, allowing themes beyond the existing competency model to emerge from the material. Inter-rater agreement reached 87%. Gwet's AC2 was 0.737, indicating substantial reliability (25). The low Fleiss' Kappa value of 0.245 reflects a known prevalence imbalance artifact in situations with high agreement but uneven category distribution (26). Full reliability statistics are reported in Supplementary material S2. The videographic analysis (23) used an independently developed grounded theory coding system and was analyzed separately from the NEOH-based qualitative content analysis.

Trustworthiness was supported through investigator triangulation, data triangulation, and preliminary member validation with participants. Investigator triangulation was achieved through independent coding by three coders. Data triangulation was achieved by combining learning journals, focus group data, and videographic observations. Preliminary member validation provided an additional check on whether the emerging interpretations resonated with participant experience. Reflexive considerations are addressed in the Constraints section.

The sample of five VPhDs represented the complete OHTC cohort and one third of the VetMedUni Vienna doctoral school's enrollment. Following Malterud et al.'s (27) information power concept, the narrow aim of the study, the high specificity of the participants, the use of multiple data sources, and the structured analysis strategy support sufficient information density despite the small number of participants. The three core patterns reported below—language discovery, communication adaptation, and transdisciplinary recognition—appeared consistently across early and late journal entries and were echoed in the focus group. This recurrence suggests thematic redundancy within this dataset, even though the study does not claim formal saturation in a broader methodological sense.

## 4 Results

Of 114 coded segments, 77 (67.5%) mapped to NEOH competencies (6) across all nine domains, while 37 (32.5%) fell outside the framework (Table 2). Three patterns dominated the mapped NEOH material: language discovery, communication adaptation, and transdisciplinary recognition. Table 3 summarizes how these and related themes appeared across cases and data sources. Where videographic data were available, convergence between self-reported and observed behavior strengthened confidence in the reported patterns.

**Table 2:** Distribution of coded segments across NEOH competency domains and inductively derived beyond-NEOH categories.

Category	*n*	%	Journal cases	FG	Key subcategories
**NEOH framework**	**77**	**67.5**			
Collective learning and reflective practice	23	29.9	SME-1, 2, 4, 5	✓	Language awareness, bidirectional learning, audience insight
Effective communication	17	22.1	SME-1, 2, 3, 4, 5	✓	Context adaptation, risk communication, visualization
Transdisciplinarity	9	11.7	SME-1, 2, 5	✓	Epistemological shift, pedagogical expertise recognition
Collaborative working	7	9.1	SME-1, 2, 3, 5	✓	Terminology negotiation, shared knowledge building
Harnessing uncertainty	6	7.8	SME-1	✓	Knowledge gaps as pedagogy, paradox communication
One Health concepts	5	6.5	SME-1, 3		OH-to-education transfer, zoonoses education
Methodological pluralism	5	6.5	SME-4	✓	Social science methods, didactic approaches
Systems understanding	3	3.9	SME-1, 4, 5		Complexity awareness, topic scope
Equity and inclusiveness	2	2.6	SME-5	✓	Knowledge heterogeneity, mutual respect
**Beyond NEOH**	**37**	**32.5**			
Implementation proposals	20	54.1	SME-1, 2, 3, 5		Network building, materials, curriculum reform
Structural barriers	7	18.9	SME-1, 2, 3, 4, 5		Curriculum rigidity, silos, resource scarcity
Contextual factors	7	18.9	SME-1, 3	✓	Teaching-research feedback, career preparation
Sustained engagement and affect	3	8.1	SME-3		Contact person role, pride, public responsibility

The table shows the distribution of the 114 coded segments across the deductively applied NEOH One Health competency domains and the inductively derived categories that did not map onto the NEOH framework. Frequencies and percentages indicate how strongly each domain or beyond-NEOH cluster was represented in the dataset.

N = 114 coded segments. Journal cases = SME cases contributing segments from learning journals. FG = focus group transcript contributed segments (✓ = yes). Focus group included 4 of 5 VPhDs (SME-2 was unable to attend). Percentages for NEOH categories calculated within 77-segment subset. SME-4 produced 2 of 3 planned journal entries. Videographic data (23) available for SME-2 and SME-3 only; not included in segment counts.

**Table 3:** Cross-case and cross-source evidence matrix for central and inductively derived themes.

Theme	SME-1 AMR/Listeria	SME-2 Beta-lacto- globulin	SME-3 Camelid dermatophytosis	SME-4 B. cereus/Organoids	SME-5 Swine/Listeria	FG (4 VPhDs; SME-2 absent)	Video (23; SME-2 + SME-3)
Language discovery	J	J + V	—	J	—	(1)	V-11
Communication adaptation	J	J + V	V	J	J	(2)	V-01–V-06
Risk communication	—	—	J + V	—	—	(2)	V-10
Epistemological shift	J	J + V	V	—	J	(3)	V-20–V-24
Uncertainty as pedagogy	J	V	V	—	—	(5)	V-37, V-38
Implementation proposals	J	J	J	—	J	—	—
Structural barriers	J	J	J	J	J	—	—
Contextual factors	J	—	J	—	—	(2)	—
Sustained engagement	—	—	J	—	—	—	—

The table summarizes how the central findings and selected inductively derived themes appeared across the five veterinary PhD student cases and data sources. It indicates whether evidence came from learning journals, the focus group, and/or available videographic observations, thereby supporting triangulation across cases and sources.

J = evidence from learning journals (verified against coded dataset, N = 114 segments). V = evidence from videographic sequences (verified against 23; 53 V-codes across SME-2 and SME-3). FG = focus group segment count in parentheses; individual speaker attributions not possible from coded transcript. SME-2 was unable to attend the focus group. Videographic data additionally evidenced Systems Understanding (V-16–V-19), Collaborative Working (V-07–V-09, V-13–V-15), One Health Concepts (V-32–V-34), Methodological Pluralism (V-35–V-36), and Equity (V-30–V-31). —, no evidence.

### 4.1 Language as a learning challenge

Collective Learning and Reflective Practice was the most frequent category (*n* = 23, 29.9%). The most salient pattern within this category was linguistic: three of the five VPhDs reported in their learning journals that they struggled to explain their research in their native language (SME-1, SME-2, and SME-4), a challenge that was also echoed in the focus group. This difficulty did not arise because the OHTC required communication in a foreign language; rather, it emerged because the VPhDs' scientific training and daily research communication were strongly shaped by English-language terminology. SME-4 (*Bacillus cereus*/organoids) reflected in her post-Meeting 1 journal:

“*You frequently use English terms because that's what you're used to from daily lab work and the literature is predominantly in English. But when you actively try to use as few English terms as possible, I had the feeling the students understood the topic well*.” (SME-4, learning journal after Meeting 1; translated from German)

SME-2 (bovine beta-lactoglobulin) similarly stated that her communication competence

“*was strengthened the most because it showed me how little I communicate about my research in my native language*.” (learning journal; translated from German)

SME-3 (camelid dermatophytosis) described the process as learning to use “*better German, not Denglisch*.” (a German portmanteau of Deutsch and Englisch, describing the habitual mixing of German and English vocabulary, also known as code-switching). In the focus group, one participant elaborated that explaining concepts in German that she had previously encountered almost exclusively in English was “*challenging*” because she was “*primarily used to* [the topic] *in English*” (focus group; translated from German).

Where videographic data were available, this self-reported language challenge was also visible in interaction. In the recorded beta-lactoglobulin-case meetings, SME-2′s explanation of beta-lactoglobulin variations in milk was characterized by pauses, reformulations, and hesitation, indicating the effort involved in translating familiar scientific concepts into accessible German (videographic sequence V-11). In the camelid dermatophytosis case, SME-3 shifted in real time from the technical term “Dermatophytosis” to the more accessible expression “skin fungal infection”, negotiating the wording collaboratively with the PSSTs (coded segment 21). To summarize, the journal entries, focus group reflections, and videographic observations suggest that the OHTC did not merely require VPhDs to simplify their research; it exposed a more fundamental linguistic transfer challenge between English-dominated scientific discourse and German-language public or educational communication.

### 4.2 Learning to communicate expertise across contexts

Effective Communication was the second most frequent category (*n* = 17, 22.1%), with evidence from all five cases and the focus group. The central finding was that communication did not appear as a generic transferable skill, but as a set of situated practices through which VPhDs learned to communicate their scientific expertise across disciplinary, educational, and public- facing contexts.

The empirical core of this category consisted of seven distinct communication practices: (1) adapting content to specific audiences, (2) translating complexity through analogies and visuals, (3) adjusting language register between scientific and everyday discourse, (4) balancing scientific precision with accessibility, (5) communicating zoonotic risk without inducing unnecessary alarm, (6) explaining through concrete examples, and (7) prioritizing content within limited time. These practices were not taught as predefined techniques. Rather, they emerged from the specific demands of interdisciplinary boundary-crossing situation between veterinary research and science education, where VPhDs had to make specialized knowledge understandable, relevant, and usable for future classroom contexts. Full subcategory definitions are provided in the Supplementary materials.

Several examples illustrate how these practices operated in context. SME-1 (AMR/Listeria) tailored the content to agricultural school students by foregrounding

“*practically relevant aspects such as cattle diseases and nutrition, marketing of animal products, and kitchen hygiene*.” (Segment 0)

This shows that effective communication required more than simplification: it involved selecting those aspects of the research that could connect meaningfully to the audience's prior knowledge and professional context. SME-4 (B. cereus/organoids) recognized that “*the topic of organoids was very new for laypeople*” and therefore required extensive visual support, including PowerPoint slides with images, to make the abstract content accessible (Segments 3, 24). The videographic data showed similar adaptations in interaction: in the camelid case, SME-3 spontaneously used hand gestures to demonstrate differences between alpaca and llama ear shapes, thereby producing a non-verbal explanation in response to the communicative situation (V-01).

Risk communication emerged as the most topic-specific of the seven practices. In the camelid dermatophytosis case, SME-3 reflected that communicating zoonotic fungal infections required avoiding unnecessary alarm: “*It shouldn't come across that you have to be afraid of llamas now*” (V-10) (cf. 22). In the focus group, one participant described the difficulty of explaining that pigs can serve as disease models for conditions they themselves do not develop, which required presenting “not knowing” as a legitimate scientific position rather than as a deficit (Segment 56). These examples show that VPhDs had to negotiate not only how much scientific information to provide, but also how to frame uncertainty, risk, and relevance responsibly.

### 4.3 “Science goes further than P-values and graphs”

Transdisciplinarity (*n* = 9, 11.7%) captured moments in which VPhDs began to recognize educational research as a legitimate knowledge system distinct from, but relevant to, their own scientific training. The central finding in this category was not simply that participants collaborated across disciplines, but that some began to reassess what counts as scientific knowledge and expertise. One participant described this as an epistemological shift in the focus group:

“*I was always in the natural sciences; that is where I was trained. This was completely new for me: that science goes further than just p-values and graphs. I was quite skeptical at first*.” (Focus group, Segment 65; translated from German)

This shift was also visible in participants' references to specific forms of pedagogical and educational knowledge that they had encountered for the first time. These included the *World Café method* (Segment 48), concept mapping as a tool for knowledge construction (Segment 25 and 32), and structured lesson planning as a systematic practice with its own disciplinary logic (Segment 64). In this sense, the VPhDs did not merely experience education as a channel for communicating already established scientific content; they encountered it as a field with its own methods, concepts, and standards of rigor. The videographic analysis of the camelid dermatophytosis case further supported this interpretation. SME-3 was observed using the knowledge of the education students to generate ideas for communicating her research topic beyond the OHTC context. This suggests an active appropriation of pedagogical expertise rather than a merely polite acknowledgment of another discipline's contribution.

Several other NEOH competency domains appeared in lower frequencies but complemented this transdisciplinary pattern. Collaborative Working (*n* = 7) was mainly reflected in terminology negotiation between disciplinary vocabularies. Harnessing Uncertainty (*n* = 6) captured moments in which VPhDs learned to communicate knowledge gaps honestly. Methodological Pluralism (*n* = 5) reflected growing appreciation for social science and educational research methods. Systems Understanding (*n* = 3) and Equity (*n* = 2) were less frequent but present; Equity appeared, for example, when one participant reflected on the need to accommodate heterogeneous prior knowledge among PSST groups (Segment 28).

Taken together, these findings suggest that the OHTC fostered more than interdisciplinary cooperation at the level of content exchange. For some VPhDs, the encounter with pre-service science teachers and educational research opened a broader understanding of science as a plural enterprise that includes pedagogical, methodological, and societal forms of knowledge.

### 4.4 Additional recommendations beyond the interview scope

Thirty-seven segments (32.5%) fell outside the NEOH framework and were distributed across four inductively derived clusters: implementation proposals, structural barriers, teaching–research feedback loops, and sustained engagement (Table 2). This was not a marginal finding. That nearly one third of the coded material did not map onto the NEOH competency framework suggests that existing One Health competency models may underspecify what emerges when health professionals engage with education as a field of practice.

The largest beyond-NEOH cluster consisted of implementation proposals (20 segments). Without being prompted, four of the five VPhDs proposed concrete strategies for sustaining One Health in schools. These included network building with teacher education institutions (Segments 86, 107, 108), the development of reusable educational materials (Segments 74, 79), Cost-effective digital learning (Segment 83), university–school partnerships (Segment 73), targeted teacher training (Segment 80), and systemic curriculum reform to promote interdisciplinarity (Segment 72). The proposals ranged from micro-level interventions, such as developing visual explainers for specific zoonoses, to macro-level suggestions, such as reforming curricula toward interdisciplinary study programs. This spontaneous strategic thinking suggests that the co-design format did not only foster communication or collaboration competencies; it also activated an orientation toward implementation and institutional change. This resonates with emerging observations that strategic action competence, although highly relevant to societal One Health impact, remains insufficiently represented in existing One Health competency frameworks (28, 29).

A second beyond-NEOH cluster concerned structural barriers (7 segments). These segments named institutional constraints that limited One Health education across all five cases. SME-2 (bovine beta-lactoglobulin) captured the core diagnosis succinctly:

“*Throughout the entire academic training, teaching happens in boxes: disciplines remain separated. Connections between concepts are rarely established*.” (Segment 89)

Other participants identified rigid curricula, time constraints, resource scarcity, and the practical difficulty that One Health spans multiple school subjects, making integration into any single subject challenging (Segment 88). These reflections indicate that the VPhDs did not only learn to communicate One Health content; they also began to diagnose the institutional conditions that make One Health education difficult to implement. This finding converges with recent work on university-level barriers to One Health training (30).

A third cluster revealed an unanticipated teaching–research feedback loop (7 segments). SME-1 (AMR/Listeria) reported that

“*practical insights from the teaching unit provide valuable guidance for my research on AMR in Listeria*.” (Segment 44)

The same participant described how simplifying complex research concepts for a non-expert audience helped them distinguish between what was essential to their research and what was unnecessary complexity that had become routine (Segment 49). In the focus group, another participant reflected that preparing for the meetings and “*always thinking about why something is done and how*” deepened their understanding of their own research rationale (Segment 45). These examples suggest a bidirectional dynamic: research informed teaching but teaching also fed back into how VPhDs understood and framed their own research. This extends existing evidence that educational outreach can benefit scientists as well as students (31) to the context of veterinary doctoral training.

Finally, three segments captured sustained engagement beyond the immediate course context. SME-3 (camelid dermatophytosis) offered to serve as a potential contact person for teachers (Segment 92) and expressed pride in contributing to didactic development (Segment 91). In the focus group, one participant, reflecting on science skepticism, described a deepened sense that communicating research to the public “*is important, that it reaches the public*” (Segment 14). Although this cluster was small, it points to a possible longer-term effect of the OHTC: participants did not only complete a course task, but in some cases began to imagine themselves as continuing contributors to science education and public engagement.

Taken together, the beyond-NEOH material suggests that the OHTC activated capacities that exceeded the deductive competency framework used for analysis. Most notably, the VPhDs generated implementation ideas, identified structural constraints, reflected on how teaching could sharpen their own research thinking, and expressed forms of sustained engagement. These findings indicate that interprofessional co-design may elicit strategic and institutional forms of action competence that are not yet fully captured in current One Health competency models.

## 5 Discussion

This community case study examined how veterinary PhD students experienced the role of subject matter experts in the OHTC, an interprofessional format that pairs doctoral researchers with pre-service science teachers to co-develop classroom materials on One Health topics. The findings suggest that the format activated competencies that conventional doctoral training does not systematically address, particularly in three areas: language awareness, communication adaptation, and transdisciplinary recognition.

The language discovery finding was unexpected. Three of the five VPhDs documented in their journals that they struggled to explain their own research in German, their native language, because their doctoral training and daily research communication were strongly shaped by English-language scientific discourse. Where videographic data were available, they supported this interpretation by showing observable hesitation, reformulation, and real-time lexical negotiation. This finding extends beyond the well-documented challenges of science communication training (32, 33) by identifying a more fundamental issue: doctoral students may lack the linguistic infrastructure to communicate their expertise outside the anglophone academic register, even in their mother tongue. The OHTC surfaced this gap not through explicit instruction, but through an encounter with an authentic audience that had an instrumental need for the content.

The communication adaptation findings resonate with recent work mapping One Health competencies onto Education for Sustainable Development frameworks. Krebser et al. (28) surveyed 28 One Health experts and identified science communication as an inductively emerging competency that extends existing Education for sustainable development frameworks, encompassing willingness to communicate with non-scientific groups, comprehensible language, and communication that encourages action. The seven communication practices identified in our data provide empirical instances of these expert-identified competencies in a doctoral training context. Notably, risk communication emerged as a distinct challenge: VPhDs had to learn to convey zoonotic risk without inducing unnecessary alarm, a competence that Heuckmann and Krüger (22) have shown requires domain-specific knowledge and that Blauza et al. (20) frame as navigating scientific uncertainty in educational settings.

The transdisciplinary recognition pattern, in which VPhDs came to view educational research as a legitimate knowledge system, is consistent with the central role that Krebser et al. (28) assign to transdisciplinarity in their One Health- Education for sustainable development competency catalog. The Synoptic Transfer Framework (19) provides a theoretical lens for this process: VPhDs moved between the scientific image of their research and the lifeworld image of the classroom, and the productive tension between these perspectives is where transdisciplinary recognition emerged. A companion study examining the pre-service teacher perspective has applied the STF hotspot structure across four thematic OHTC cases (7); generating broader empirical evidence for the framework's applicability through videographic interaction analysis across institutions and countries remains a priority for future research. The challenge VPhDs faced in communicating scientific uncertainty without undermining trust further illustrates the STF's relevance for understanding how scientific findings can inform, but not dictate, educational and societal responses.

That 32.5% of coded segments fell outside the NEOH framework warrants attention. The 20 unprompted implementation proposals suggest that the participatory format activated what Krebser et al. (28) term strategic action competence, which their expert survey identified as central to societal One Health impact but which existing One Health competency frameworks inadequately address. The VPhDs did not merely acquire competencies; they spontaneously proposed how to sustain the connection between research and education at systemic, institutional, and material levels. This finding is consistent with evidence that educational outreach benefits scientists themselves (31) and extends it by showing that doctoral students, when placed in a co-design format, can generate implementation knowledge that neither didactic training nor isolated workshops typically produce.

### 5.1 Limitations

Several limitations should be considered when interpreting these findings. The sample was small (*n* = 5), although it represented the complete OHTC cohort and one third of the doctoral school. Accordingly, the findings should not be generalized without replication across additional cohorts, institutions, and national contexts. Data density also varied across participants: SME-1 contributed 40 of the 84 journal-derived segments, while the other participants contributed between 9 and 14 segments each; SME-4 completed only two of the three planned journal entries. To assess the influence of this imbalance, we conducted a sensitivity check excluding all SME-1 segments. The three core patterns persisted, and the category ranking remained stable, with Collective Learning and Effective Communication retaining their first and second positions. The category most affected was Transdisciplinarity, which decreased from 11.7 to 6.9%.

The analysis focused only on the VPhD perspective. The PSST experience is reported elsewhere (7). The focus group was conducted approximately one semester after course completion and included four of the five VPhDs, as SME-2 was unable to attend. Because individual speaker attributions were not possible from the coded focus group transcript, focus group findings are reported collectively. Videographic data were available for only two of the five cases, namely SME-2 and SME-3. The videographic analysis (23) is currently available as an unpublished master's thesis; efforts are underway to make it accessible through the HAUP institutional repository.

Finally, the study did not include pre-post measurement and therefore cannot establish causal effects. The self-report data may also be affected by social desirability bias. In addition, the deductive use of the NEOH framework may have directed analytic attention toward expected competency domains. However, the inductive coding of segments that did not fit the framework partially mitigated this limitation and enabled the identification of beyond-NEOH themes.

### 5.2 Practical recommendations

For institutions seeking to replicate or adapt the OHTC, three design elements appear particularly consequential. First, the format should provide an authentic audience with an instrumental need for the scientific content. In the OHTC, PSSTs did not engage with the VPhDs' research as passive recipients or evaluators; they needed to understand it well enough to transform it into classroom materials and teach it in schools. This created a communication context that differed substantially from simulated science communication exercises. Rubega et al. (32) found no significant audience-perceived difference between trained and untrained graduate student communicators when audiences served primarily as evaluators. In contrast, the OHTC suggests that audience authenticity and task dependency may be important conditions for meaningful science communication learning.

Second, reciprocal dependency between the two groups appears to be a structural driver of engagement. VPhDs depended on PSSTs to translate their research into pedagogically meaningful classroom formats, while PSSTs depended on VPhDs to ensure scientific accuracy and relevance. This mutual dependency reflects the co-learning, co-designing, co-creating, and co-sharing logic associated with Two-Eyed Seeing approaches, grounded in respect, responsibility, and reciprocity (Hogue and Provost, 2023, cited in 19).

Third, the STF hotspot mapping (Table 1) provides a replicable structure for sequencing interprofessional encounters. The iterative movement between Engagement Contexts, Scientific Integrity, Scientific Application, and Narrative Shaping helped organize the collaboration without reducing it to a linear transfer model. Institutions without teacher education partners could adapt the format by collaborating with other non-health disciplines or professional groups that require accurate scientific content to be translated for specific public, educational, or community-facing audiences, including settings such as physician–patient communication where complex health information must be made accessible and actionable for non-specialist recipients.

## 6 Conclusion

This community case study shows that the OHTC can create learning conditions that conventional veterinary doctoral training rarely provides. By placing VPhDs in a reciprocal co-design process with pre-service science teachers, the format required doctoral expertise to become not only scientifically accurate, but also pedagogically usable and socially meaningful.

The study's central contribution is to show that such learning does not emerge from generic communication training alone. It emerged from an authentic interdisciplinary boundary-crossing situation in which doctoral researchers had to negotiate language, audience, uncertainty, educational expertise, and implementation possibilities. The fact that nearly one third of coded material fell outside the NEOH framework further suggests that interprofessional co-design may activate strategic and institutional action competence that current One Health competency models do not yet fully capture.

These findings contribute to the emerging evidence base on interprofessional One Health education and illustrate how the Synoptic Transfer Framework can structure encounters in which scientific expertise becomes communicable, teachable, and actionable in practice. The results remain exploratory and require replication across cohorts, institutions, and national contexts. Nevertheless, the study indicates that authentic collaboration between veterinary researchers and education professionals can help doctoral students move beyond producing One Health knowledge toward enabling its use in society.

## Data Availability

The raw data supporting the conclusions of this article will be made available by the authors, without undue reservation.
