# Supplementary material for: The One Health teaching clinic as an interprofessional education learning environment: an Austrian community case study of veterinary PhD students' competency development through co-design with pre-service science teachers
Source: Front Vet Sci. 2026 Aug 28;13:1781542. doi: 10.3389/fvets.2026.1781542 (PMC13565391; doi:10.3389/fvets.2026.1781542)
Supplement: Supplementary file 1 [file Data_Sheet_1.docx]

**Supplementary Materials**

# S1. Coding Manual

Coding followed qualitative content analysis (Kuckartz & Rädiker, 2023) in two phases. In the deductive phase, the nine NEOH One Health Core Competencies (Laing et al., 2023) served as main categories. In the inductive phase, segments that did not fit any NEOH category were assigned to emergent categories developed from the data. The coding manual below presents each category with its definition and decision rules used by the three independent coders.

## S1.1 Deductive Categories: NEOH Competency Framework

| **Code** | **NEOH Competency** | **Domain** | **Coding Rule** |
| --- | --- | --- | --- |
| 1 | Effective Communication | Skills | Segments describing communication strategies, audience adaptation, language modification, use of examples/visuals, risk communication, or reflection on communicative challenges in the SME–PSST interaction. |
| 2 | Collaborative and Resilient Working | Skills | Segments describing joint work, shared goal-setting, negotiation of terminology, building shared knowledge bases, or constructive handling of challenges in the collaboration. |
| 3 | Systems Understanding | Skills | Segments demonstrating awareness of interconnections between domains (human, animal, environmental health), complexity layers, or systemic relationships in research content or educational design. |
| 4 | Transdisciplinarity | Values | Segments describing engagement with or recognition of knowledge systems outside one’s own discipline, particularly pedagogical/didactic expertise. Includes silo-breaking and cross-disciplinary integration. |
| 5 | Social, Cultural and Gender Equity and Inclusiveness | Values | Segments addressing heterogeneous prior knowledge, inclusive communication strategies, or recognition of diverse learner backgrounds. |
| 6 | Collective Learning and Reflective Practice | Values | Segments describing self-reflection on professional habits, language awareness, iterative learning, perspective-taking, recognition of own knowledge gaps, or bidirectional learning processes. |
| 7 | One Health Concepts | Knowledge | Segments demonstrating understanding of OH principles, OH-to-education transfer, or zoonotic disease education rationale. |
| 8 | Theoretical and Methodological Pluralism | Knowledge | Segments recognizing or engaging with methodological approaches outside natural sciences, particularly social science and educational research methods. |
| 9 | Harnessing Uncertainty, Paradox and Limited Knowledge | Knowledge | Segments describing communication of uncertainty, knowledge gaps, paradoxical research situations, or the nature of science as a process. |

## S1.2 Inductive Categories

Segments that could not be assigned to any of the nine NEOH competencies were coded as “Not Applicable” and subsequently organized into emergent subcategories through discursive agreement among three coders. Four meta-categories emerged:

**Implementation Solutions:** Concrete proposals for sustaining One Health education, including network building, material development, teacher training, curriculum reform, digital resources, and institutional support.

**Structural Barriers:** Institutional constraints impeding OH integration, including curriculum rigidity, disciplinary silos, resource scarcity, time constraints, and subject separation.

**Contextual Factors:** Dynamics specific to the doctoral-teacher setting, including teaching-research feedback, career preparation, science communication in society, and research-practice bridging.

**Sustained Engagement & Affect:** Willingness to continue beyond the course, personal pride, and sense of public responsibility.

# S2. Inter-Rater Reliability Statistics

Three independent coders (SK, SL, FM) coded all 114 text segments. None of the coders was involved in course design or delivery. The principal investigator (UH) adjudicated disagreements and reviewed emergent categories.

## S2.1 Agreement Metrics

| **Metric** | **Value** | **Interpretation** |
| --- | --- | --- |
| Overall percentage agreement | 87% | Proportion of segments with identical codes across coders |
| Gwet’s AC2 | 0.737 | Substantial agreement (Landis & Koch, 1977) |
| Fleiss’ Kappa | 0.245 | See note on kappa paradox below |

## S2.2 Note on the Kappa Paradox

The low Fleiss’ Kappa (0.245) despite high percentage agreement (87%) is a well-documented statistical artifact known as the kappa paradox (Gwet, 2008). When one category dominates the distribution—in this case, Collective Learning and Reflective Practice accounted for 29.9% of coded segments—kappa statistics are deflated because the high baseline probability of agreement by chance reduces the denominator of the kappa formula. Gwet’s AC2 was designed specifically to address this limitation and provides a more accurate estimate of inter-rater reliability under conditions of prevalence imbalance. The value of 0.737 falls in the “substantial agreement” range according to the widely used benchmarks of Landis and Koch (1977), which were originally developed for Cohen’s Kappa but are routinely applied to Gwet’s AC2 in the methodological literature.

# S3. NEOH Competency Distribution

Of 114 coded segments, 77 (67.5%) were assigned to NEOH competency categories with coder agreement. The distribution is shown below.

| **Competency** | **Domain** | **n** | **%** | **Key Finding** |
| --- | --- | --- | --- | --- |
| Effective Communication | SKILLS | 17 | 22.1% | Second most frequent - central to PhD-teacher interaction |
| Collaborative and Resilient Working | SKILLS | 7 | 9.1% |  |
| Systems Understanding | SKILLS | 3 | 3.9% |  |
| Transdisciplinarity | VALUES AND ATTITUDES | 9 | 11.7% | Actively recognized and valued |
| Social, Cultural and Gender Equity and Inclusiveness | VALUES AND ATTITUDES | 2 | 2.6% |  |
| Collective Learning and Reflective Practice | VALUES AND ATTITUDES | 23 | 29.9% | Dominant competency - strong reflective practice |
| One Health Concepts | KNOWLEDGE AND AWARENESS | 5 | 6.5% |  |
| Theoretical and Methodological Pluralism | KNOWLEDGE AND AWARENESS | 5 | 6.5% |  |
| Harnessing Uncertainty, Paradox and Limited Knowledge | KNOWLEDGE AND AWARENESS | 6 | 7.8% |  |

# S4. Emergent Categories Beyond NEOH Framework

Thirty-seven segments (32.5%) did not fit the NEOH framework and were coded inductively. The full list of emergent subcategories is presented below.

| **Subcategory** | **n** | **Meta-Category** | **Significance** |
| --- | --- | --- | --- |
| Emergent Category | Frequency | Category Type | Significance for OHTC Setting |
| SOLUTION_Network_Building | 3 | Implementation Solutions | Building educational sector networks |
| SOLUTION_Educational_Materials | 2 | Implementation Solutions | Development of didactic materials |
| SUSTAIN_Continued_Engagement | 2 | Sustainability | Willingness for future collaboration |
| CONTEXT_Science_Communication_Society | 1 | Societal Context | Addresses science skepticism - missing in NEOH |
| CONTEXT_Teaching_Research_Feedback | 1 | Research Impact | Bidirectional learning: teaching informs research |
| CONTEXT_Public_Engagement | 1 | Public Engagement | Research acceptance through youth involvement |
| CONTEXT_Research_Practice_Bridge | 1 | Research-Practice Transfer | Core mechanism of OHTC setting |
| CONTEXT_Curriculum_Integration | 1 | Structural | Curriculum placement considerations |
| BARRIER_Time_Constraints | 1 | Structural Barriers | Rigid curriculum time limitations |
| BARRIER_Political_Resources | 1 | Structural Barriers | Need for political decisions on resources |
| SOLUTION_Curriculum_Flexibility | 1 | Implementation Solutions | Elective courses as alternative |
| SOLUTION_Systemic_Reform | 1 | Implementation Solutions | Curriculum reform for interdisciplinarity |
| SOLUTION_School_Partnerships | 1 | Implementation Solutions | University-school partnerships |
| SOLUTION_Institutional_Support | 1 | Implementation Solutions | Embedding in curricula and competency catalogs |
| SOLUTION_Project_Integration | 1 | Implementation Solutions | Integration into existing school projects |
| SOLUTION_Research_School_Cooperation | 1 | Implementation Solutions | Systematic university-school cooperation |
| SOLUTION_Sustainability_Strategy | 1 | Implementation Solutions | Short and long-term sustainability strategies |
| SOLUTION_Teacher_Training | 1 | Implementation Solutions | Targeted teacher professional development |
| BARRIER_Resource_Scarcity | 1 | Structural Barriers | Lack of resources for new approaches |
| SOLUTION_Interdisciplinary_Units | 1 | Implementation Solutions | Cross-subject teaching units |
| SOLUTION_Digital_Resources | 1 | Implementation Solutions | Cost-effective digital platforms |
| SOLUTION_Curriculum_Integration | 1 | Implementation Solutions | Systematic curriculum integration |
| SOLUTION_Comprehensive_Strategy | 1 | Implementation Solutions | Multi-faceted approach |
| BARRIER_Curriculum_Rigidity | 1 | Structural Barriers | Inflexible curricula prevent innovation |
| BARRIER_Multidisciplinary_Scope | 1 | Structural Barriers | OH spans multiple subjects - integration difficult |
| BARRIER_Disciplinary_Silos | 1 | Structural Barriers | Education organized in disciplinary boxes |

# S5. Complete Coded Segment Table (N = 114)

The table below presents all 114 coded text segments with segment ID, source document, NEOH category assignment, subcategory code, and a brief coding rationale. Source documents are abbreviated: ANTIBI = AMR/Listeria journals (SME-1); RINDER = Bovine beta-lactoglobulin journals (SME-2); ALPAKA = Camelid dermatophytosis journals (SME-3, with number indicating journal entry); BACILL = Food pathogens/organoids journals (SME-4); SCHWEI = Swine health journals (SME-5); FG = Focus group transcript.

| **ID** | **Source** | **NEOH Category** | **Subcategory** | **Coding Rationale (excerpt)** |
| --- | --- | --- | --- | --- |
| 0 | ANTIBI | Effective Communication | COMM_Context_Adaptation | Tailoring the communication explicitly to the target audience (agricultural students) using practical examples from their own lives |
| 1 | ANTIBI | Systems Understanding | SYS_Complexity_Awareness | Understanding of levels of complexity (from simplified to detailed) and their interrelationships within the scientific system |
| 2 | ANTIBI | Effective Communication | COMM_Complexity_Translation | Learning to communicate complex scientific topics in an accessible way – a key element of effective science communication |
| 3 | BACILL | Effective Communication | COMM_Visualization_Strategy | Use of visual aids (PowerPoint, images) to aid understanding – a teaching strategy |
| 4 | FG | Collective Learning and Reflective Practice | REFL_Bidirectional_Learning | Reflections on two-way learning – PhD students recognize that teacher training students already have more experience, |
| 5 | ANTIBI | One Health Concepts | OH_Education_Transfer | Integrating One Health into the education sector – applying and transferring One Health concepts to an educational context |
| 6 | ANTIBI | Transdisciplinarity | TRANS_Competence_Development | Developing interdisciplinary skills through collaboration – a key aspect of transdisciplinarity |
| 7 | ANTIBI | Effective Communication | COMM_Simplification_Tools | Specific communication strategies: analogies, practical examples, and illustrations designed to be accessible to non-specialists |
| 8 | ANTIBI | Effective Communication | COMM_Language_Adaptation | Awareness of the need to adapt language and to avoid or explain technical jargon |
| 9 | RINDER | Effective Communication | COMM_Precision_vs_Accessibility | Striking a balance between precision (technical terms) and clarity (explanations) – an important communication skill |
| 10 | SCHWEI | Effective Communication | COMM_Example_Based | Specific examples from the target group’s everyday lives to help overcome communication barriers |
| 11 | SCHWEI | Effective Communication | COMM_Prior_Knowledge_Adaptation | Tailoring communication to prior knowledge and adopting an inclusive approach (excluding no one) |
| 12 | FG | Effective Communication | COMM_Professional_Transfer | Transferring communication skills to veterinary practice (communication with pet owners) – practical relevance |
| 13 | FG | Effective Communication | COMM_Risk_Communication | Sensitive risk communication regarding zoonoses – providing information without causing panic, a key aspect of public health communication skills |
| 14 | FG | Not Applicable | CONTEXT_Science_Communication_Society | Social context: skepticism towards science and the importance of science education – goes beyond the NEOH framework |
| 15 | FG | Effective Communication | COMM_Risk_Communication | See Section 13 – Risk communication regarding zoonoses |
| 16 | FG | Effective Communication | COMM_Content_Prioritization | Learning to focus on what matters – a key communication and teaching skill |
| 17 | ANTIBI | Transdisciplinarity | TRANS_Competence_Development | See Section 6 – Interdisciplinary Skills Development |
| 18 | ANTIBI | One Health Concepts | OH_Education_Transfer | See Section 5 – Operational budget transfers in the education sector |
| 19 | BACILL | Collective Learning and Reflective Practice | REFL_Language_Awareness | Reflection on one’s own language habits (English vs. German) in an academic context |
| 20 | RINDER | Collective Learning and Reflective Practice | REFL_Mothertongue_Gap | Key consideration: Recognizing a lack of communication skills in one’s native language – specifically in a PhD context |
| 21 | ALPAKA-3 | Collaborative and Resilient Working | COLL_Terminology_Negotiation | Collaborative discussion of specialist terminology across disciplines – two-way collaboration |
| 22 | ANTIBI | Harnessing Uncertainty, Paradox and Limited Knowledge | UNCERT_Misconception_Recognition | Dealing with misunderstandings and limited knowledge among learners – making productive use of uncertainty |
| 23 | BACILL | Systems Understanding | SYS_Topic_Scope_Understanding | An understanding of the varying scope and focus of different groups |
| 24 | BACILL | Effective Communication | COMM_Visualization_Strategy | A renewed emphasis on visual explanation strategies |
| 25 | FG | Collective Learning and Reflective Practice | REFL_Iterative_Refinement | An iterative learning process through dialogue – collective learning and adaptation |
| 26 | FG | Collective Learning and Reflective Practice | REFL_Abstraction_Recognition | Reflection on the level of abstraction in one’s own research and the need for a clearer explanation |
| 27 | FG | Harnessing Uncertainty, Paradox and Limited Knowledge | UNCERT_Paradox_Communication | Communicating paradoxical situations (pigs as a model for a disease they do not contract) |
| 28 | FG | Social, Cultural and Gender Equity and Inclusiveness | EQUITY_Knowledge_Heterogeneity | Dealing with heterogeneity of knowledge and the need for inclusive approaches |
| 29 | ALPAKA-2 | One Health Concepts | OH_Zoonoses_Education | Communicating zoonoses as a core concept of public health to the public |
| 30 | BACILL | Collective Learning and Reflective Practice | REFL_Audience_Interest | Reflection on public interest and a focus on relevant topics |
| 31 | RINDER | Effective Communication | COMM_Multi_Level_Juggling | The complexity of communication: assessing prior knowledge, using clear language and remaining precise all at the same time |
| 32 | RINDER | Collective Learning and Reflective Practice | REFL_Perspective_Taking | Perspective-taking: What is viewed positively in research may be perceived negatively outside it |
| 33 | FG | Collective Learning and Reflective Practice | REFL_Soft_Skills_Development | Reflection on personal and soft skills development through participation |
| 34 | FG | Collective Learning and Reflective Practice | REFL_Knowledge_Breadth_Awareness | Becoming aware of the extent of one’s own knowledge and the fact that it cannot be taken for granted |
| 35 | FG | Transdisciplinarity | TRANS_Active_Emphasis | A deliberate focus on interdisciplinarity in OH topics (foodborne pathogen resistance) |
| 36 | FG | Collaborative and Resilient Working | COLL_Purpose_Understanding | The need for a shared understanding to ensure effective cooperation |
| 37 | FG | Collective Learning and Reflective Practice | REFL_Peer_Learning | Learning about other PhD projects through exchange – collaborative learning among PhD students |
| 38 | FG | Collective Learning and Reflective Practice | REFL_Soft_Skills_Development | See Section 33 – Soft Skills and Broadening Horizons |
| 39 | FG | Collective Learning and Reflective Practice | REFL_Understanding_Importance | Reflection on the importance of understanding among students |
| 40 | ALPAKA-2 | One Health Concepts | OH_Zoonoses_Education | See section 29 |
| 41 | ALPAKA-4 | Effective Communication | COMM_Risk_Communication | Raising awareness of the need for careful communication regarding zoonotic risks |
| 42 | RINDER | Effective Communication | COMM_Language_Switch_Challenge | The challenge of language conversion (English to German) and avoiding misinterpretations |
| 43 | SCHWEI | Collective Learning and Reflective Practice | REFL_Knowledge_Distribution | Reflections on the distribution of knowledge and miscommunication between departments |
| 44 | ANTIBI | Not Applicable | CONTEXT_Teaching_Research_Feedback | Two-way transfer: Teaching experiences provide insights for one’s own AMR research |
| 45 | FG | Collective Learning and Reflective Practice | REFL_Research_Rationale | Reflection on one’s own research rationale through the teaching process |
| 46 | FG | Theoretical and Methodological Pluralism | PLUR_Pedagogical_Methods | Exploring teaching methods as a new methodological approach |
| 47 | FG | Collective Learning and Reflective Practice | REFL_Student_Capability | Reflection on trainee teachers’ skills and one’s own learning outcomes |
| 48 | BACILL | Theoretical and Methodological Pluralism | PLUR_Pedagogical_Methods | Learning new teaching methods (World Café) |
| 49 | ANTIBI | Collective Learning and Reflective Practice | REFL_Simplification_Without_Loss | Reflections on simplifying complex concepts without losing depth |
| 50 | SCHWEI | Collaborative and Resilient Working | COLL_Cross_Field_Adaptation | Learning how to adapt scientific information for other disciplines |
| 51 | BACILL | Collective Learning and Reflective Practice | REFL_Language_Awareness | Reflections on the importance of standard German academic language versus “Denglish” |
| 52 | FG | Collective Learning and Reflective Practice | REFL_Mothertongue_Gap | Recognizing one’s own gaps in knowledge of German technical terms |
| 53 | FG | Harnessing Uncertainty, Paradox and Limited Knowledge | UNCERT_Comfortable_Not_Knowing | Dealing with one’s own uncertainty and realizing that some details are not essential |
| 54 | FG | Harnessing Uncertainty, Paradox and Limited Knowledge | UNCERT_Knowledge_Gaps_Communication | Communication regarding a limited knowledge base and early stages of research |
| 55 | FG | Harnessing Uncertainty, Paradox and Limited Knowledge | UNCERT_Knowledge_Gaps_Communication | Explaining research gaps to the public |
| 56 | FG | Harnessing Uncertainty, Paradox and Limited Knowledge | UNCERT_Process_Nature | Conveying that uncertainty and the fact that “there is no answer” are part of the scientific process |
| 57 | FG | Transdisciplinarity | TRANS_OH_Core_Principle | An explicit understanding of transdisciplinarity as a core principle of OH |
| 58 | ALPAKA-1 | Not Applicable | CONTEXT_Public_Engagement | The importance of public engagement and research acceptance—broader than NEOH |
| 59 | ANTIBI | Transdisciplinarity | TRANS_Multiple_Disciplines | Incorporation of multiple disciplines (education, sociology, environmental science) for OH |
| 60 | ANTIBI | Transdisciplinarity | TRANS_Perspective_Integration | Integrating various perspectives (biomedical, social, and educational) |
| 61 | ANTIBI | Transdisciplinarity | TRANS_Beyond_Monodiscipline | The realization that AMR cannot be viewed solely from a scientific perspective |
| 62 | RINDER | Transdisciplinarity | TRANS_Different_Approaches | Appreciation of different approaches in education |
| 63 | FG | Theoretical and Methodological Pluralism | PLUR_Social_Science_Methods | Introduction to social science approaches and methods |
| 64 | FG | Theoretical and Methodological Pluralism | PLUR_Pedagogical_Methods | Didactic knowledge as a new methodological perspective |
| 65 | FG | Theoretical and Methodological Pluralism | PLUR_Epistemological_Shift | Engaging with fundamentally different ways of thinking between the natural sciences and the social sciences |
| 66 | ANTIBI | Not Applicable | CONTEXT_Research_Practice_Bridge | Research-Practice Transfer as a distinct category |
| 67 | FG | Not Applicable | CONTEXT_Curriculum_Integration | Considerations on Integrating Overhead Projector Topics into the Curriculum - Structural Level |
| 68 | ALPAKA-1 | Collaborative and Resilient Working | COLL_Institutional_Commitment | The need for institutional willingness to cooperate |
| 69 | ALPAKA-1 | Not Applicable | BARRIER_Time_Constraints | Structural barrier: Time constraints due to rigid curricula |
| 70 | ALPAKA-1 | Not Applicable | BARRIER_Political_Resources | Structural barrier: Human resources require political decisions |
| 71 | ALPAKA-3 | Not Applicable | SOLUTION_Curriculum_Flexibility | Proposed solution: Electives as an alternative to the required curriculum |
| 72 | ANTIBI | Not Applicable | SOLUTION_Systemic_Reform | Proposed Solution: Systemic Curriculum Reform to Promote Interdisciplinarity |
| 73 | ANTIBI | Not Applicable | SOLUTION_School_Partnerships | Proposed solution: Establishing school-university partnerships |
| 74 | ANTIBI | Not Applicable | SOLUTION_Educational_Materials | Proposed solution: Development of educational materials |
| 75 | ANTIBI | Not Applicable | SOLUTION_Institutional_Support | Proposed solution: Incorporation into curricula and competency catalogs |
| 76 | ANTIBI | Not Applicable | SOLUTION_Project_Integration | Proposed solution: Integrate OH into interdisciplinary school projects |
| 77 | ANTIBI | Not Applicable | SOLUTION_Research_School_Cooperation | Proposed Solution: Systematic University-School Partnerships |
| 78 | ANTIBI | Not Applicable | SOLUTION_Sustainability_Strategy | Proposed Solution: Short- and Long-Term Sustainability Strategies |
| 79 | ANTIBI | Not Applicable | SOLUTION_Educational_Materials | Proposed solution: Provision of educational materials |
| 80 | ANTIBI | Not Applicable | SOLUTION_Teacher_Training | Proposed solution: Targeted teacher training |
| 81 | ANTIBI | Not Applicable | BARRIER_Resource_Scarcity | Structural barrier: Lack of resources as an obstacle |
| 82 | ANTIBI | Not Applicable | SOLUTION_Interdisciplinary_Units | Proposed Solution: Development of Interdisciplinary Lesson Plans |
| 83 | ANTIBI | Not Applicable | SOLUTION_Digital_Resources | Proposed solution: Cost-effective digital learning platforms |
| 84 | ANTIBI | Not Applicable | SOLUTION_Curriculum_Integration | Proposed solution: Systematic integration into the curriculum |
| 85 | ANTIBI | Not Applicable | SOLUTION_Comprehensive_Strategy | Proposed Solution: Comprehensive Strategy (Professional Development + Materials + Interdisciplinarity) |
| 86 | ANTIBI | Not Applicable | SOLUTION_Network_Building | Proposed solution: Establishing a network with teacher education programs |
| 87 | BACILL | Not Applicable | BARRIER_Curriculum_Rigidity | Structural barrier: Rigid curricula prevent the inclusion of new topics |
| 88 | RINDER | Not Applicable | BARRIER_Multidisciplinary_Scope | Structural barrier: OH encompasses multiple disciplines, making it difficult to integrate |
| 89 | RINDER | Not Applicable | BARRIER_Disciplinary_Silos | Structural barrier: Education in disciplinary “boxes” |
| 90 | SCHWEI | Not Applicable | BARRIER_Subject_Separation | Structural barrier: Discipline-based curriculum |
| 91 | ALPAKA-1 | Not Applicable | AFFECT_Personal_Pride | Affective dimension: Pride in one's own contribution—the emotional component |
| 92 | ALPAKA-1 | Not Applicable | SUSTAIN_Continued_Engagement | Sustainability: Willingness to collaborate in the future |
| 93 | ALPAKA-1 | Not Applicable | SUSTAIN_Continued_Engagement | Sustainability: Openness to future collaboration |
| 94 | ALPAKA-2 | Not Applicable | CONTEXT_Career_Preparation | Career Preparation: Training for Future Knowledge Transfer Tasks |
| 95 | ANTIBI | Collective Learning and Reflective Practice | REFL_External_Perspective | Reflection through an outside perspective: “Taking a step back” offers a new perspective |
| 96 | ANTIBI | One Health Concepts | OH_Pedagogical_Principles | OH instruction based on specific pedagogical principles (participatory, practice-oriented) |
| 97 | ANTIBI | Collective Learning and Reflective Practice | REFL_Feedback_Integration | The Importance of Feedback in Interdisciplinary Fields |
| 98 | ANTIBI | Not Applicable | CONTEXT_Research_Impact | Research Impact: Educational Experiences Deepen Research and Enhance Its Relevance |
| 99 | BACILL | Collective Learning and Reflective Practice | REFL_Audience_Interest | Reflecting on Student Interest Through Feedback |
| 100 | SCHWEI | Collective Learning and Reflective Practice | REFL_Public_Knowledge | Learning about the general public's “basic knowledge” through student feedback |
| 101 | RINDER | Collaborative and Resilient Working | COLL_Terminology_Negotiation | The Need for Technical Terminology Harmonization in Interdisciplinary Collaboration |
| 102 | SCHWEI | Social, Cultural and Gender Equity and Inclusiveness | EQUITY_Mutual_Respect | Mutual acceptance and respect for different levels of knowledge |
| 103 | ALPAKA-3 | Collaborative and Resilient Working | COLL_Expert_Pedagogue_Synergy | The necessary collaboration between subject matter experts and educators, and the resulting synergistic effects |
| 104 | ANTIBI | Collaborative and Resilient Working | COLL_Cross_Disciplinary_Communication | Collaboration for Better Outreach and Wider Public Awareness |
| 105 | ANTIBI | Effective Communication | COMM_Audience_Adaptation | Tailoring research communication to different audiences |
| 106 | ANTIBI | Not Applicable | SOLUTION_Science_Education_Cooperation | Proposed Solution: Structured Collaboration Between Science and Education |
| 107 | ANTIBI | Not Applicable | SOLUTION_Network_Building | Proposed Solution: Interdisciplinary Networks |
| 108 | ANTIBI | Not Applicable | SOLUTION_Network_Building | Proposed Solution: Long-Term Networking |
| 109 | RINDER | Not Applicable | SOLUTION_Active_Exchange | Proposed Solution: Active Exchange Between Education and Science for Knowledge Transfer |
| 110 | RINDER | Collective Learning and Reflective Practice | REFL_Feedback_Integration | Feedback mechanism for updating course content |
| 111 | SCHWEI | Systems Understanding | SYS_Interconnected_Domains | Understanding OH as a bridge between previously separate fields |
| 112 | SCHWEI | Transdisciplinarity | TRANS_Silo_Breaking | The explicit importance of breaking down disciplinary silos |
| 113 | SCHWEI | Not Applicable | SOLUTION_Workshop_Format | Proposed Solution: Workshops as a Format for Research-to-School Transfer |

# S6. Videographic Analysis Methodology

Videographic recordings of the SME–PSST online meetings were available for two of the five cases: the camelid dermatophytosis case (SME-3; meetings 1, 2, and 3 of Group A recorded; meetings of Group B partially recorded) and the bovine beta-lactoglobulin case (SME-2; meetings 2 and 3 of Group A recorded). For the remaining three cases (SME-1 AMR/Listeria, SME-4 food pathogens, SME-5 swine/Listeria epidemiology), no video recordings were available or analyzable within the evaluation timeframe.

The videographic analysis was conducted as part of a master’s thesis (Fraisl, 2025) using Grounded Theory methodology (Huber & Lehmann, 2016). A coding system was developed iteratively from the video material, independent of the NEOH-based content analysis. Sequences were coded into the following categories:

| **Category** | **Description and Examples** |
| --- | --- |
| Communication Methods | Observable use of analogies, visual aids, non-verbal communication, targeted questions, or structured explanations (e.g., V-01: demonstrating ear shape with hand gestures; V-04: using structured verbal transitions) |
| Adapted Communication | Observable modification of language register, checking for understanding, explaining technical terms in lay language (e.g., V-02: asking “Was anything unclear?”; V-03: explaining “risk group” with examples) |
| Own Perspective | SME makes their reasoning or interpretation explicit during interaction (e.g., V-05: disclosing uncertainty about terminology; V-06: respectfully disagreeing about didactic method) |
| Challenges, Flexibility, Adaptability | Observable difficulties in communication, pausing, stuttering, or actively navigating challenges (e.g., V-10: sensitively framing zoonotic risk; V-11: stuttering during German explanation of beta-lactoglobulin; V-15: reflecting on miscommunication) |
| Social Behaviors, Emotions, Relationship Building | Expressing empathy, giving positive feedback, showing interest, acknowledging others’ contributions (e.g., V-13: expressing appreciation; V-14: showing empathy for frustrating teaching experience) |
| Shared Knowledge Base | Observable construction of common understanding between SME and PSST groups through explanation, negotiation, and verification |

The convergence between the videographic categories and the NEOH-based content analysis provided methodological triangulation. Both independently developed coding systems identified communication adaptation, collaborative knowledge building, and reflective self-awareness as core processes. Specific sequences referenced in the main manuscript (V-01, V-10, V-11, V-15) were verified against the original video recordings and the Fraisl (2025) analysis.

## S6.1 Case Selection and Sampling of Videographic Data

Recordings were made of the SME–PSST online meetings held between November 2024 and February 2025. Because the complete corpus exceeded what could be transcribed and analysed within the timeframe of the master’s thesis, a purposive selection of two topic cases was made (Fraisl, 2025). Excerpts of all available recordings were first screened. Cases were then selected to maximise contrast, so that the influence of any individual SME’s communicative style on the observed patterns would be reduced. The selection criteria were: (1) differences in speaking time across the meeting, (2) the amount of observable collaborative interaction, and (3) differences in communicative behaviour. On this basis, the camelid dermatophytosis case (SME-3; Groups A and B) and the bovine beta-lactoglobulin case (SME-2; Group A) were selected. This sampling strategy means that the videographic material illustrates contrasting cases rather than a representative cross-section of the cohort, and videographic findings are therefore reported in the main manuscript as triangulating rather than primary evidence.

## S6.2 Transcription Conventions

The videographic analysis was based on complete transcriptions of the selected recordings. The following conventions were applied (Fraisl, 2025). They are reproduced here because the markers for pausing, hesitation and self-interruption underpin the observations on language adaptation reported in Section 4.1 of the main manuscript (e.g., sequence V-11).

| **Convention** | **Rule applied** |
| --- | --- |
| Timestamps | Recorded at every change of speaker in the format 00:00:00, placed on the same line as the speaker identifier and the utterance. |
| Speaker anonymisation | Speakers are identified only as “Speaker 1” to “Speaker 7”. Numbering remains consistent within each group across all three meetings. No names or other identifying details are retained in the transcripts. |
| Line breaks | A new line begins at each change of speaker. |
| Completeness and dialect | Utterances are transcribed in full; dialect is rendered in comprehensible standard German. |
| Pauses, fillers, hesitation | Noticeable pauses, filler words, or hesitant speech are noted in parentheses preceding the relevant passage. Where fillers and linguistic difficulties accumulate to an extent that noticeably affects the communication, they are transcribed in full. |
| Interruptions | Noticeable interruptions, whether self-induced or induced by another speaker, are marked with a hyphen (-). |
| Unintelligible passages | Marked as (unintelligible). |
| Non-verbal communication | Recorded in parentheses where it is made explicit through verbal cues. |
| Closing sequences | Concluding farewells are either summarised as (farewells) or transcribed in full. |

## S6.3 Anchor Sequences Referenced in the Main Manuscript

The table below lists the videographic sequences cited in the main manuscript, with the case, the observation, and the inductively developed subcategory assigned in the original analysis (Fraisl, 2025). Sequence identifiers correspond to the numbering used in that analysis. Quotations are English translations of the German originals; positions refer to line numbers in the transcripts.

| **ID** | **Case and meeting** | **Observation** | **Subcategory (Fraisl, 2025) and interpretation** |
| --- | --- | --- | --- |
| V-01 | SME-3, camelids, Group B, Meeting 1 | “This is how an alpaca looks and this is how a llama looks” – ear shape demonstrated with the hands. | Communication methods: demonstrates ear shape to distinguish the species, adding a non-verbal method to the verbal explanation. |
| V-02 | SME-3, camelids, Group A, Meeting 1 | “Do you have any questions, was anything unclear? About what you read? Or do you want to know more about anything?” | Adapted communication: asks specifically about the pre-service teachers’ comprehension. |
| V-03 | SME-3, camelids, Group A, Meeting 1 | The term “risk group” is explained using examples. | Adapted communication: explains the technical term with practice-oriented examples. |
| V-04 | SME-3, camelids, Group B, Meeting 1 | Structured verbal transition from general species information to the doctoral topic, summarising content through questions. | Communication methods: uses a clear linguistic transition and summarises content through questions. |
| V-05 | SME-2, beta-lactoglobulin, Group A, Meeting 2 | “It is easier, because at first I thought, ‘more frequently’?” | Presentation of own perspective: makes her reading of the wording in the teaching materials, and the possible misunderstandings, explicit. |
| V-06 | SME-2, beta-lactoglobulin, Group A, Meeting 3 | In a discussion about the value of copying from slides, the SME disagrees with a pre-service teacher: “To improve spelling, I would say yes.” | Presentation of own perspective: disagreement in discussion shows that her own view is communicated transparently. |
| V-10 | SME-3, camelids, Group A, Meeting 2 | “… because it should not come across that you now have to be afraid of llamas. That is a bit the danger of this topic, but that people are made somewhat aware.” | Challenges, flexibility, adaptability: potentially misleading communication and its negative consequences are perceived as a challenge. |
| V-11 | SME-2, beta-lactoglobulin, Group A, Meeting 2 | The explanation of the differing occurrence of beta-lactoglobulin in milk was marked by stuttering and pauses. | Challenges, flexibility, adaptability: difficulties in the subject-matter explanation surface as linguistic difficulties. |
| V-13 | SME-3, camelids, Group A, Meeting 3 | “Yes, and you have the One Health topic and this defusing afterwards. I don’t know, it is really perfect.” | Social behaviours, emotions, relationship building: positive feedback on the preparation and delivery of the lesson. |
| V-14 | SME-2, beta-lactoglobulin, Group A, Meeting 3 | “I think that was a bit frustrating for you too, wasn’t it? When you plan a lesson for so long and then the pupils don’t want to.” | Social behaviours, emotions, relationship building: understanding and empathy for disappointing experiences. |
| V-15 | SME-2, beta-lactoglobulin, Group A, Meeting 3 | “For me it was that it is sometimes difficult. Ehm. To express oneself in such a way that it is then also interpreted that way …” | Challenges, flexibility, adaptability: the perceived challenge of being misunderstood is addressed; difficulties in communication are simultaneously observable. |
| V-22 | SME-3, camelids, Group A, Meeting 2 | “Maybe, just as a tip, I think ‘dermatophytosis’ is too difficult for pupils. I would use ‘skin fungus’ as the term … you understand it immediately, don’t you?” | Inter- and transdisciplinary working, thinking, behaviour: actively contributes to a task developed within the discipline of didactics; corresponds to the register shift described in Section 4.1 of the main manuscript. |

## S6.4 Consent, Data Protection and Availability

All participants were informed at the outset that the meetings would be recorded and actively consented to the recording and to its subsequent analysis. This consent did not extend to the publication of the raw recordings. Because each recording documents an interaction between one SME and several pre-service science teachers, and not all participants consented to publication, the raw video material cannot be shared or made available on request. Consent covers analysis and reporting in anonymised and aggregated form only.

Analytic transparency is therefore established through the derived materials rather than through the recordings themselves. The complete videographic coding system, the transcription conventions, the case selection criteria, and the anchor sequences underlying every videographic claim made in the main manuscript are reproduced in full in Sections S6 and S6.1 to S6.3 of these Supplementary Materials. No videographic finding reported in the manuscript rests on material that is withheld here: each cited sequence is listed with its case, meeting, observation, and assigned subcategory, so that the inferential chain from observation to interpretation can be examined independently of the recordings. Transcripts were anonymised at the point of transcription (see S6.2); personal data were removed and speakers were identified by number only. Further aggregated and anonymised material from the underlying analysis, including the full coding scheme and the anonymised transcript excerpts, is available from the corresponding author on reasonable request. The underlying master's thesis (Fraisl, 2025) is held at the library of the University College for Agricultural and Environmental Education (HAUP) and catalogued in the Austrian Library Network (<https://permalink.obvsg.at/AC17804633>), where it is available for consultation.

*Note.* The three core patterns reported in the main manuscript — language discovery, communication adaptation, and transdisciplinary recognition — rest on the learning journals and the focus group, which were available for all five cases. The videographic material corroborates these patterns for the two recorded cases but does not carry any finding on its own.

# S7. Participant Overview

| **Identifier** | **Research Topic** | **Doctoral Year** | **PSST Groups** | **Data Sources Available** |
| --- | --- | --- | --- | --- |
| SME-1 | AMR in Listeria (livestock) | ≥2nd year | 2 groups | Journals (3 entries) + Focus group |
| SME-2 | Bovine beta-lactoglobulin | ≥2nd year | 2 groups | Journals (3 entries) + Focus group + Videography |
| SME-3 | Zoonotic fungal infections (camelids) | ≥2nd year | 2 groups | Journals (3 entries) + Focus group + Videography |
| SME-4 | Bacterial food pathogens / organoids | ≥2nd year | 2 groups | Journals (2 entries) + Focus group |
| SME-5 | Swine health | ≥2nd year | 2 groups | Journals (3 entries) + Focus group |

*Note.* SME-1 contributed 40 of 84 journal-derived segments; SME-4 produced only 2 of 3 planned journal entries (no third entry or closing reflection). Videographic recordings were available for SME-2 (meetings 2 and 3 of one group) and SME-3 (meetings 1, 2, and 3 of one group; partial recordings of second group). Four of five VPhDs participated in the focus group (SME-2 was unable to attend).

# S8. Cross-Case Analysis Methodology

Table 3 in the main manuscript presents a cross-case pattern analysis mapping key themes against the five SME cases and available data sources. The construction followed these rules:

**Attribution criterion:** A theme was attributed to a specific case when at least one coded segment from that case’s journal data or a clearly attributable focus group statement documented the theme. For the focus group (FG), individual statements were attributed to specific SMEs based on speaker identification in the transcript. Where a theme appeared only in the focus group discussion without clear individual attribution, it was marked as FG for all participants who contributed to that discussion exchange.

**Videographic evidence:** Videographic sequences (V-codes from Fraisl, 2025) were added as a separate column where recorded interaction sequences corroborated a self-reported pattern. Video data were available for SME-2 (beta-lactoglobulin case, Meetings 2 and 3 of Group A) and SME-3 (camelid case, Meetings 1–3 of Group A, partial Group B). The three remaining cases (SME-1, SME-4, SME-5) could not be videographically corroborated.

**Interpretation:** Cells marked with J (journal) indicate individual written reflection; FG indicates the focus group transcript contributed segments to this theme. Individual speaker attributions within the focus group are not possible from the coded transcript, as speakers were not identified. A dash (—) indicates no evidence from that case’s data for this theme. Absence of evidence should not be interpreted as absence of the phenomenon; it may reflect variation in journal depth (SME-1 produced 40 segments vs. 9–14 for others) or the specific content of the meetings that were or were not recorded.

# S9. Learning Journal Prompts

SMEs received structured journal prompts at three timepoints during the semester. The prompts were designed to elicit reflection on the collaboration experience and were provided in German. Below are English translations of the core prompts.

**Journal Entry 1 (Week 4, after Meeting 1):**

(1) How did you experience the first meeting with the pre-service teachers? What surprised you? (2) How did you find communicating your research topic to a non-specialist audience? What was easy, what was difficult? (3) What did you learn about your own communication through this experience?

**Journal Entry 2 (Week 9, after Meeting 2):**

(1) How did you experience reviewing the pre-service teachers’ lesson plans? Were you surprised by how they translated your research? (2) What did you learn from seeing your research topic through a pedagogical lens? (3) How has the collaboration influenced your thinking about communication and/or interdisciplinary work?

**Journal Entry 3 (Week 13, closing reflection):**

(1) Looking back at the entire semester: what was the most important experience for you? (2) Which of your competencies do you feel were strengthened through the OHTC? Which were not? (3) What would you change about the format? What suggestions do you have for future iterations? (4) Do you see connections between this experience and your future professional practice?

*Note.* The structured nature of these prompts should be considered when interpreting the findings. Prompts 1.2, 1.3, 2.3, and 3.2 explicitly direct attention to communication and competencies, which partly explains the prominence of these themes in the coded data. The emergent categories (implementation proposals, structural barriers, teaching-research feedback) arose despite the prompt structure rather than because of it, lending additional weight to these findings.
